# The impact of the WHO Framework Convention on Tobacco Control in defending legal challenges to tobacco control measures

**DOI:** 10.1136/tobaccocontrol-2018-054329

**Published:** 2018-06-02

**Authors:** Suzanne Y Zhou, Jonathan D Liberman, Evita Ricafort

**Affiliations:** 1 McCabe Centre for Law and Cancer, Melbourne, Victoria, Australia; 2 McCabe Centre for Law and Cancer, Manila, Philippines

**Keywords:** litigation, tobacco industry, public policy, global health, human rights

## Abstract

**Background:**

Since the WHO Framework Convention on Tobacco Control’s (FCTC) entry into force, the tobacco industry has initiated litigation challenging tobacco control measures implemented by governments around the world, or supported others to initiate such litigation on its behalf. In defending their tobacco control measures against such litigation, governments have invoked their obligations and rights under the WHO FCTC. We assess the extent to which the WHO FCTC has provided legal weight to governments’ defences against legal challenge.

**Methods:**

We reviewed 96 court decisions concerning legal challenges to tobacco control measures, determining whether or not they cited the WHO FCTC and their outcomes. We then reviewed the cases where the WHO FCTC was cited, analysing how the WHO FCTC contributed to the resolution of the case.

**Results:**

The WHO FCTC was cited in 45 decisions. Decisions both citing and not citing the WHO FCTC were largely decided in favour of governments, with 80% of WHO-FCTC-citing and 67% of non-WHO-FCTC-citing cases upholding the measure in its entirety and on every ground of challenge. In cases where it was cited, the WHO FCTC contributed to the resolution of the case in favour of governments by providing a legal basis for measures, demonstrating the measure’s public health purpose, demonstrating the evidence in favour of a measure, demonstrating international consensus, demonstrating that a measure promotes or protects health-related human rights and demonstrating whether or not a measure is reasonable, proportionate or justifiable.

**Conclusions:**

The way the WHO FCTC has been cited in court decisions suggests that it has made a substantial contribution to courts’ reasoning in tobacco control legal challenges and has strengthened governments’ arguments in defending litigation.

## Introduction

Since the entry into force of the WHO Framework Convention on Tobacco Control (WHO FCTC), the tobacco industry has initiated and supported litigation challenging various tobacco control measures around the world, in order to prevent, delay or weaken their implementation. This has included challenges under trade and investment law, such as the cases brought against Australia^1 2^ and Uruguay’s^3^ tobacco packaging and labelling laws, and challenges in domestic and regional courts.^4–7^ Due to the litigious nature of the tobacco industry, legal challenges are commonly cited as a barrier to the implementation of strong tobacco control measures.^8 9^

Many responding governments invoke the WHO FCTC when they defend against such challenges in court.^4^ Building on a report prepared as evidence to inform the WHO FCTC Impact Assessment Expert Group’s report to the seventh session of the Conference of the Parties (COP7),^10 11^ this paper looks at the contributions such invocations make to the successful defence of legal challenges, by reviewing cases in which the WHO FCTC has been cited in decided judgements, and analysing its contribution to the court or tribunal’s reasoning. Such analysis is important because successful defences ultimately affect the chances of tobacco control measures being fully implemented or kept in place, can give confidence to other countries looking to implement similar measures in the face of actual and/or threatened litigation, and contribute to a body of comparative case law that can help policymakers evaluate and respond to legal arguments that arise during the policy development process. The contribution of the WHO FCTC to such defences is therefore an important aspect of its contribution to the implementation of tobacco control measures, and complements its impact on the adoption of measures by legislators or policymakers, analysed in other papers in this special supplement.

## Methodology

To identify cases where the WHO FCTC was invoked by governments in defending measures implementing the WHO FCTC against litigation, we reviewed cases under:

- The ‘legal challenges’ tag of www.tobaccocontrollaws.org, a database managed by the Campaign for Tobacco Free Kids covering tobacco control case law in 60 jurisdictions (297 cases reviewed for relevance).^12^
- Cases featured in the McCabe Centre Knowledge Hub website, a resource on key legal challenges to WHO FCTC implementation hosted at www.untobaccocontrol.org/kh/legal-challenges (25 cases reviewed for relevance).^13^
- All cases in the ‘tobacco’ tag of the Global Health and Human Rights Database (globalheathrights.org), which covers public health-related cases invoking constitutional or international human rights law across 119 jurisdictions (51 cases reviewed for relevance).^14^

All three databases cover both common and civil law jurisdictions and include cases in languages other than English. In total, the review was conducted across a set of 331 cases after removing duplicates, and covers a time period spanning 27 February 2005 (the entry into force date of the WHO FCTC) to 1 December 2017.

In reviewing the cases, our inclusion criteria were: the respondent had to be party to the WHO FCTC at the time of the decision, and the case needed to be a legal challenge aimed at striking down or hindering the full implementation of a tobacco control measure, rather than a challenge seeking to compel a higher standard of implementation. The paper focuses on challenges to regulatory measures, so we excluded challenges solely about the application of a law to individual companies or persons (as opposed to challenges to the validity of the law itself), and enforcement proceedings. We included cases which had been appealed (with judgements on the merits at all stages of the litigation included), but removed preliminary or solely procedural orders unless they resulted in the final dismissal of a case. This gave us a set of 96 judgements in 26 jurisdictions meeting the inclusion criteria. We then divided the set by whether or not the court’s judgement cited the WHO FCTC—45 judgements across 20 jurisdictions did so. Online supplementary table 1 summarises the facts, outcomes and WHO FCTC-related aspects of these 45 cases. Online supplementary table 2 summarises more briefly the 96 cases meeting the inclusion criteria.

10.1136/tobaccocontrol-2018-054329.supp1 Supplementary data

10.1136/tobaccocontrol-2018-054329.supp2 Supplementary data

The cases were reviewed against the inclusion criteria based on the summaries and metadata provided in the relevant databases (with some cases reviewed against full judgements if summaries were not available or were unclear). We determined whether judgements cited the WHO FCTC by searching full judgements and translations in English for ‘Framework’, ‘Convention’ or ‘FCTC’; full judgements in Spanish for ‘convenio’, ‘marco’ and ‘CMCT’; and full judgements in French for ‘convention’, ‘cadre’ and ‘CCLAT’. For other languages, we searched machine translations into English, including additionally the terms ‘obligation’, ‘international’ and ‘treaty’ to take into account the possibility of translation differences. We read in full all cases that cited the WHO FCTC and were either in English, or had a human translation into English, to determine how the WHO FCTC was used in the case, covering a total of 29 cases, and reviewed a combination of summaries and machine translations for the remaining 16 cases.

The limitations of this study include that we were only able to fully review cases that were in English, or had an English translation. Most cases in languages other than English were reviewed based on unofficial translations without the assistance of certified translation services. We relied heavily on database-provided summaries in reviewing whether cases met the inclusion criteria, and in reviewing cases in other languages.

## Results

### Overall outcomes (and their limitations)

Of the 45 judgements citing the WHO FCTC, 36 (80%) were decided fully in favour of the respondent government; two cases upheld but modified the relevant measure; six were decided in favour of the claimant (four on the grounds that the measure should have been implemented by legislation rather than decree/regulation, one based on consistency with a regional standard, and one on the grounds that a smoke-free law made unjustified distinctions between different types of dining establishments); and one was an advisory opinion on the relevant legal principles that was then sent to another court for resolution on the facts. Of the 51 cases not citing the WHO FCTC, 34 (67%) were decided fully in favour of respondent governments, 3 upheld but modified the measure, 1 upheld one measure and invalidated another and 13 were decided in favour of the claimant (two of which were later decided in favour of the government on appeal). In both groups, most litigation was brought by the tobacco industry, although there were also cases brought by individual smokers,^i^ retail establishments,^ii^ legislators,^iii^ filmmakers^iv^ and other governments or government/regulatory agencies.^v^ Overall, the WHO FCTC was more likely to be cited in the defence of measures which were the subject of a detailed or precise recommendation in the WHO FCTC or its guidelines at the time (such as smoke-free laws, graphic health warnings, plain packaging, flavour bans, pack size requirements and restrictions on tobacco advertising, promotion and sponsorship, including retail display bans), while cases not citing the WHO FCTC were often about measures for which the WHO FCTC and its guidelines were less prescriptive (eg, the inclusion of mental health institutions and prisons in smoke-free laws), although this was not uniform and citation patterns were also heavily influenced by national differences in judicial writing styles (such as differences in the length and detail of judgements expected in a particular jurisdiction).

Although the outcome data above might superficially suggest that judgements citing the WHO FCTC are more likely to uphold a challenged measure than judgements which do not, on their own, the figures say relatively little about the WHO FCTC’s impact. Since the databases we used generally require cases to be selected, reviewed, translated and summarised, the kinds of cases included in each database may reflect prioritisation by the database editors, rather than (or as well as) underlying patterns about the kinds of litigation that are occurring and their outcomes. National practices relating to joining or consolidating cases can skew the numbers of cases in each category—what is multiple dockets in one jurisdiction may be one consolidated proceeding in another, which means that the numbers of cases in each category depend significantly on how they are counted. It is also possible that judges upholding a measure are more likely to cite the WHO FCTC than judges striking down a measure. Finally, each case is decided on its own facts, and their outcomes often turn on relatively subtle differences, which means that it is not possible to draw broad conclusions from win/lose outcomes alone.

As such, we considered *how* the WHO FCTC was used in the cases where it was invoked, rather than simply the overall outcomes. We discuss the results of this analysis below, which should be presumed to be illustrative, not exhaustive—a series of case studies, not a ‘dataset’.

### Contribution of the WHO FCTC and its guidelines to court decisions

The WHO FCTC contributed to the defence of a measure in various ways where cited. These included:

#### As a legal basis for a measure

Many courts emphasised that the WHO FCTC provided a legal basis for a measure,^vi^ particularly where tobacco control measures were challenged for being outside the power of the legislature or implementing agency.

In the Kenyan case of *British American Tobacco v Cabinet Secretary for Health*,^15^ for example, the Court of Appeal of Kenya considered a challenge to the Kenyan Tobacco Control Regulations, a comprehensive suite of tobacco control measures which included a requirement that tobacco companies pay a compensatory contribution to a tobacco control fund. This provision was challenged by the tobacco industry as being ‘an attempt to irregularly apply the FCTC’ (among other challenges to a range of different provisions on different grounds). In rejecting the challenge to the compensation requirement, the Court stated that ‘the enactment of the Tobacco Act… can only be viewed as an attempt to fulfil [the] obligation’ in article 3 of the WHO FCTC, ‘to implement measures to protect its present and future generations from the devastating social and environmental consequences of tobacco consumption and exposure to tobacco smoke’. It stated that as an international treaty binding Kenya, the WHO FCTC formed part of Kenyan law under article 2 (6) of Kenya’s Constitution, and that therefore ‘the enactment of the Tobacco Act and Tobacco Regulations are anchored on the Constitution of Kenya and no inconsistency arises.’

In the case of *Ceylon Tobacco v Minister of Health*,^16^ which concerned a challenge to Sri Lanka’s large graphic health warnings, the Sri Lankan Court of Appeal considered WHO FCTC article 11 and its guidelines in concluding that legislation that authorised ‘health warnings’ covered both text and graphic health warnings, noting that:

Our Supreme Court in decided cases emphasized the need to interpret domestic law in harmony with Sri Lanka’s commitments even in cases where no specific domestic law had been enacted… Having read FCTC and the guidelines for implementing of Article 11 of the FCTC there cannot be any prohibition to convey the message by pictorial health warnings … Our courts recognize international commitments and [relevant articles] of the Constitution endeavor to foster respect for international law and treaty obligation.

In a legislative consultation brought by 10 lawmakers to the Constitutional Division of the Supreme Court of Costa Rica (No 2012-0 03918)^17^ questioning the constitutionality of a tobacco control law, the Court noted that the legislation implemented the WHO FCTC. It upheld the law in its entirety, considering in particular that WHO FCTC articles 6, 8, 13 and 16 provided a legal basis for provisions introducing a specific excise tax on cigarettes; prohibiting smoking in enclosed public places; banning tobacco advertising, promotion and sponsorship; and raising the minimum pack size from 10 to 20 sticks.

#### Demonstrating that a measure has a public health purpose

The WHO FCTC was also important in a number of cases for demonstrating that a measure had a bona fide public health purpose and therefore fell within countries’ rights and powers to regulate for public health.^vii^ For example, in *Philip Morris v Uruguay*, the arbitral tribunal cited the fact that Uruguay’s graphic health warnings and single presentation requirement implemented the WHO FCTC in finding that they were public health measures implemented in light of Uruguay’s sovereign right to regulate.^3^ In Costa Rica, after noting that the challenged tobacco control law implemented the WHO FCTC, the Court cited the WHO FCTC preamble and concluded that ‘there is no doubt that the measures questioned in the enquiry are in keeping with the objective assumed by our country to place effective restrictions on tobacco, all with the goal of protecting public health.’^17^

#### As evidentiary support

The WHO FCTC, and in particular, its guidelines and other COP decisions, were used to support the evidence base in favour of a measure in several cases.^viii^

For example, in *Philip Morris Norway v Health and Care Services of Norway*, the Oslo District Court in Norway found that it was ‘accepted knowledge’ that tobacco advertising, promotion and sponsorship led to increased consumption of tobacco products, as reflected in WHO FCTC article 13, and cited the article 13 guidelines in finding that display of products at point of sale constituted tobacco advertising, promotion and sponsorship and that a ban on such display was a suitable measure for protecting public health.^18^ Courts in Canada, the UK and Colombia cited the article 13 guidelines in reaching similar conclusions about the evidence base for a retail display ban in Nova Scotia (*R v Mader’s Tobacco Store* 19), a vending machine ban in the UK (*Sinclair Collis v Secretary of State for Health* 20) and a comprehensive tobacco, advertising and sponsorship ban in Colombia (C-830/10).^21^ In *Poland v EU*, the European Court of Justice noted, while hearing a challenge to a ban on characterising flavours, that the WHO FCTC guidelines for articles 9 and 10 should be ‘of particularly high evidential value’, given their basis in the ‘best available scientific evidence’.^22^ In *Philip Morris v Uruguay*, the arbitral tribunal held that Uruguay was entitled to rely on evidence available at the international level and on scientific and technical cooperation through the WHO FCTC, and was not required to conduct local studies in introducing its measures to implement article 11.^3^

Article 5.3 of the WHO FCTC and its guidelines have also been cited to demonstrate the need to exercise caution in relation to evidence generated by the tobacco industry.^ix^ In assessing evidence introduced into court by tobacco companies to challenge standardised packaging laws in the UK, the High Court of England and Wales remarked that: ‘The question of the intrinsic quality of the evidence is a fundamental one, not least because of Article 5 (3) FCTC and the WHO guidelines… to the effect that the tobacco industry should be treated as having adopted a deliberate policy of subverting public health policy through, inter alia, the deployment of its substantial capital and organisational resources to generate evidence designed to contradict the established policy consensus.’^23^ The Court of Appeal affirmed the High Court’s use of article 5.3 and its guidelines, noting that the Court ‘was entitled to treat them as telling in favour of subjecting the evidence of the tobacco companies to rigorous scrutiny.’^24^

#### Demonstrating international consensus

A related use of the WHO FCTC was to demonstrate international consensus in relation to the need for a measure and the nature of the problem it addresses.^x^ In *BAT South Africa v Minister for Health*, for example, an unsuccessful challenge to an advertising ban on the basis of freedom of commercial expression, the Supreme Court of Appeal of South Africa noted that ‘South Africa … has international law obligations to ban tobacco advertising and promotion, and that this has been the practice in many other open and democratic societies.’^25^ In *JTI v Canada* (Attorney-General), in which tobacco companies unsuccessfully challenged an advertising ban and requirement for graphic health warnings covering 50% of the principal display areas of tobacco packs, the Supreme Court of Canada noted that:

Governments around the world are implementing anti-tobacco measures similar to and, in some cases, more restrictive than Canada’s… The WHO Framework Convention stipulates that warning labels ‘should’ cover at least 50 percent and ‘shall’ cover at least 30 percent of the package.^26^

In *Philip Morris v Uruguay*, the tribunal noted that the graphic health warnings implemented by Uruguay were based on the ‘internationally accepted’ principle in article 11 and its guidelines that large health warnings should be mandated on tobacco product packaging.^3^

The WHO FCTC has also been used to demonstrate consensus about the seriousness of tobacco use as a public health problem.^xi^ In C-830/10, the Colombian Constitutional Court cited the WHO FCTC to demonstrate that there was a global consensus that tobacco consumption had serious consequences for health and the environment.^21^ In *BAT v Secretary of State for Health*, the High Court of England and Wales stated that:

The FCTC contains at its heart two propositions of real significance for the present case. The first is that tobacco use is an ‘epidemic’ of global proportions which exerts a catastrophic impact upon health… The second… is that the tobacco companies have over multiple decades set out, deliberately and knowingly, to subvert attempts by government around the world to curb tobacco use and promote public health.^23^

#### Demonstrating that a measure promotes or protects human rights

In certain countries, the WHO FCTC was cited to elaborate on state duties to promote and protect constitutional rights to health, life or a healthy environment.^xii^ This has often been used to counter legal challenges based on (or ostensibly based on) other rights or freedoms, such as constitutional protections for commercial speech or property.

For example, in a Peruvian case filed by 5000 individuals against a ban on smoking in public places,^27^ the Constitutional Court of Peru found that the WHO FCTC was a human rights treaty elaborating how the constitutional right to health was to be implemented, and was therefore a treaty with constitutional rank to be taken into account in assessing the challenge to the law. The court found that the contribution of the smoke-free law to the constitutionally obligatory aim of realising the right to health outweighed the relatively minor incursion on personal autonomy and freedom of commerce involved in regulating where smoking could take place. The Constitutional Court of the Former Yugoslav Republic of Macedonia, in upholding a ban on smoking in public places,^28^ found that the WHO FCTC gave content to the right of everyone to the enjoyment of the highest attainable standard of physical and mental health enshrined in article 12 of the International Covenant on Economic, Social and Cultural Rights.^29^ The link between WHO FCTC implementation and human rights was also recognised in cases in Colombia,^21^ Guatemala,^30^ Panama,^31^ South Africa^25^ and Uruguay.^3^ In each of these cases, the contribution of the WHO FCTC to fundamental rights in relation to health was considered to be a strong reason for the limitation of the interests invoked by claimants.

#### As a benchmark for reasonableness, proportionality or justifiability

Finally, the WHO FCTC was often cited to demonstrate that a measure was reasonable, proportionate or justified, whether in the context of demonstrating that it meets the requirements of exceptions and limitations to particular commercial rights, or in the context of stand-alone requirements that a measure be proportional to its objectives.^xiii^ This overlaps with the use of the WHO FCTC to show bona fide objectives, evidence, international commitments, or the existence of competing rights or interests. However, a number of judicial statements discuss more generally how the WHO FCTC supports findings that measures are reasonable.

To give a few examples, the South African Supreme Court of Appeal in *BAT South Africa v Minister of Health* found that the fact that a comprehensive advertising ban implemented article 13 of the WHO FCTC provided a ‘compelling case’ that the limitation on tobacco companies’ freedom of commercial expression was justified for public health reasons, and stated that it was ‘obliged, under the Constitution, to give weight to [the WHO FCTC] in determining the question of justification or the limitation of the right to freedom of speech.’^25^ The European Court of Justice, in dismissing a challenge to the 2014 EU Tobacco Products Directive, noted that ‘the EU legislature cannot be accused of having acted arbitrarily in selecting a figure of 65% for the area reserved for combined health warnings… that selection is based on criteria deriving from the FCTC recommendations and, in making it, the EU legislature acted within the bounds of its broad discretion.’^32^ In *Philip Morris v Uruguay*, the arbitral tribunal noted that the WHO FCTC could be used as a ‘point of reference for the reasonableness’ of Uruguay’s measures, and referred to the WHO FCTC extensively in its findings that Uruguay’s measures were reasonable and therefore did not breach obligations to provide fair and equitable treatment to foreign investors.^3^

## Discussion

The above results show the many ways in which the WHO FCTC has strengthened respondent governments’ defences to legal challenges. For the most part, it is difficult to ‘prove’ that the WHO FCTC was directly responsible for the outcomes of any particular case. There is no counterfactual for what would have happened if the WHO FCTC were not in force. Further, many cases are decided on multiple grounds, each of which may be sufficient on its own to dismiss a challenge, and not all of which involve the WHO FCTC. To take one example, the argument in *Philip Morris v Uruguay* that Uruguay’s tobacco packaging measures expropriated Philip Morris’ investments was dismissed both on the grounds that there was no substantial deprivation of Philip Morris’ investments, where the WHO FCTC was not relevant, and on the grounds that the measures were an exercise of Uruguay’s sovereign right to regulate, where the WHO FCTC was critical to the resolution of the question.^3^ It is possible that judgements invoking the WHO FCTC might invoke other arguments to reach the same outcome were the WHO FCTC never concluded.

However, what the results do show is that the WHO FCTC is extensively relied on by parties in defending cases and by courts in reaching their conclusions, and that it contributes to the reasoning of the latter in important ways. In particular, the WHO FCTC plays a major role in translating a large and sometimes complex body of scientific evidence into a format that is comprehensible to legal institutions and assimilable to legal concepts, as indicated by the use of the WHO FCTC to support evidentiary claims, show international consensus in support of a measure, establish a public health purpose and demonstrate reasonableness. In this respect, the role of the WHO FCTC guidelines has been crucial—as a key source of guidance to parties on good practices and the best available scientific evidence, and in showing courts that parties have based their measures on such. In many instances, it is likely that the evidence before the court might have been more contested or complex in the absence of the WHO FCTC and its guidelines—which may not have affected the outcome of the case, but would almost certainly have placed heavier (and costlier) burdens on those defending the measure.

Additionally, in many countries, the WHO FCTC has influenced the legal framework for the implementation of tobacco control measures, either by providing a basis for governments’ authority to act, or through providing guidance on the interpretation or application of constitutional provisions on human rights or public health. The scope of government powers and duties to protect public health, and the weight they should be given relative to other powers and duties (such as those relating to commercial activities) are at the heart of many legal challenges to tobacco control measures, and it is evident from the cases that both the inclusion of tobacco control within public health powers and duties and the weight that is given to tobacco control measures (whether independently or by way of the right to health, life or a healthy environment) are strengthened by their inclusion in a binding international treaty.

Our findings have implications for broader debates on how to address the risk of future legal challenges, particularly in the context of concerns about the use of trade and investment law by the tobacco industry. Many proposals to address the risk of legal challenges focus specifically on the relationship between international trade and investment and the WHO FCTC, including through trade and investment treaty reform^33^ and/or actions by the WHO FCTC COP to manage potential conflict across treaty regimes.^34^ However, while trade and investment specific work is important, our findings suggest that there is also much to be gained from strengthening the WHO FCTC and its implementation overall. In particular, elaborating on the substance and rationale of the obligations (particularly through guidelines and other normative materials) and providing guidance on how they might apply to emerging areas of tobacco control assist parties in adopting effective tobacco control measures, and in defending them in court. Meanwhile, establishing linkages between WHO FCTC implementation and broader issue areas such as sustainable development and human rights is particularly important given the number of cases where challenges are resolved on human rights grounds; or where courts must consider the scope, limits and purposes of protections for economic activity.

Finally, it is worth noting that citations in decided judgements are a relatively narrow indicator of impact, and do not capture other ways in which the WHO FCTC might have influenced the outcomes of litigation, for example, through better multisectoral coordination in drafting and defending litigation, cooperation and assistance from other parties, or strengthened political will to defend cases.

## Conclusions

Overall, our review shows that many courts have extensively relied on the WHO FCTC to support their conclusions to dismiss legal challenges against WHO FCTC-implementing measures. The WHO FCTC has been invoked in a wide variety of ways, both as a binding legal instrument and as a source of technical and scientific authority. The WHO FCTC has supported findings of the efficacy and reasonableness of tobacco regulation, helped to more sharply define the interests at stake and triggered more judicial scrutiny of arguments made by the tobacco industry. This suggests that the WHO FCTC has made a substantial contribution to the practice of courts in the consideration of tobacco control litigation, and has been an essential part of many governments’ legal strategy for defending measures.

**What this paper adds**

- Previous research indicates that tobacco control measures are frequently challenged in court, that this is a barrier to the implementation of effective tobacco control measures and that most of these challenges have little merit.
- The WHO Framework Convention on Tobacco Control (FCTC) has often been cited by courts in resolving these challenges, but studies have not examined the different ways in which the WHO FCTC contributes to the resolution of the case, and few studies on legal challenges to tobacco control measures examine challenges under all three of domestic, regional and international laws.

- Our review provides a qualitative assessment of how the WHO FCTC contributes to the reasoning of courts when resolving legal challenges against tobacco control measures, across domestic, regional and international laws.
- It demonstrates that the WHO FCTC has been invoked in a variety of ways to support successful defences against legal challenges, including by strengthening the legal and evidentiary basis of measures, demonstrating bona fide public health purpose, demonstrating international consensus, supporting arguments based on human rights and demonstrating the reasonableness of a measure.

## Footnotes

i See online supplementary table 2, lines 1, 12, 24, 32, 35, 50, 65, 73, 74, 86, 87, 89, 91, 92. References to examples in tables are non-exhaustive.

ii See online supplementary table 2, lines 15, 19, 21, 49, 54, 67, 78, 81, 84.

iii See online supplementary table 2, lines 20, 45.

iv See online supplementary table 2, lines 28, 82, 88.

v See online supplementary table 2, lines 56, 68, 79.

vi See online supplementary table 1, lines 1, 9, 17, 23, 33, 35, 41, 42, 45.

vii See online supplementary table 1, lines 4, 21, 23, 34, 35, 39, 45.

viii See online supplementary table 1, lines 4, 6, 7, 18, 19, 21, 27, 29, 30, 42.

ix See online supplementary table 1, lines 2, 5.

x See online supplementary table 1, lines 4, 22, 44.

xi See online supplementary table 1 lines 1, 2, 5, 30, 40.

xii See online supplementary table 1, lines 4, 9, 12, 13, 15, 22, 23, 24, 26, 30, 34, 37, 39, 42— see also examples on right to health, below  No   38.

xiii See online supplementary table 1, lines 2, 4, 5, 6, 7, 12, 13, 14, 15, 16, 21, 22, 42, 44.
