# Supplementary material for: The impact of the WHO Framework Convention on Tobacco Control in defending legal challenges to tobacco control measures
Source: Tob Control. 2018 Jun 2;28(Suppl 2):s113–8. doi: 10.1136/tobaccocontrol-2018-054329 (PMC6589463; doi:10.1136/tobaccocontrol-2018-054329)
Supplement: Supplementary data [file tobaccocontrol-2018-054329supp001.docx]

**Table 1: legal challenges to regulatory measures in WHO FCTC parties – judgments which cite the WHO FCTC**

| Title | Respondent country / court | Decision date | Measure challenged | Grounds of challenge | Outcome | How was FCTC cited or invoked? |
| --- | --- | --- | --- | --- | --- | --- |
| 1. British American Tobacco Ltd v. Ministry of Health, CA No. 112 of 2016 (2017) | Kenya (Court of Appeal) | February 17, 2017 | Tobacco control regulations (including smoke-free laws, tobacco packaging and labelling, disclosure requirements, article 5.3 implementation, and requirements on tobacco companies to contribute 2% of value of products manufactured to a compensation fund) | Public participation rights, due process, legal basis, discrimination, privacy, intellectual property, interpretation of FCTC | Regulations upheld in entirety | To provide a legal basis for certain measures, as indication of the nature of the tobacco industry, indication of the negative impacts of tobacco use, demonstrating that tobacco is not like other products |
| 1. BAT v. Secretary of State for Health [2016] EWCA Civ 1182 | United Kingdom (Court of Appeal of England and Wales) | November 30, 2016 | Standardised (plain) packaging | Intellectual property, proportionality, property and other commercial rights, consistency with EU Tobacco Products Directive | Measure upheld | Demonstrating proportionality of measure, demonstrating need to exercise caution in relation to tobacco industry evidence |
| 1. British American Tobacco Panama S.A. s/ Executive Decree 611 of 2010, Docket Nos. 788-10, 818-10, 1013-10.* | Panama (Supreme Court) | August 03, 2016 | Decree extending legislative ban on tobacco advertising, promotion, and sponsorship to retail display | Intellectual property, consumer right to receive information | Measure upheld | FCTC article 13 guidelines used to interpret scope of Panama’s obligations regarding tobacco advertising, promotion and sponsorship (TAPS) |
| 1. Philip Morris SÀRL v. Uruguay, ICSID Case No. ARB/10/7 | Uruguay (ad hoc investment tribunal) | July 08, 2016 | 80% graphic health warnings (GHWs), ban on brand variants | Intellectual property, investment law – fair and equitable treatment, expropriation, due process rights, umbrella clause | Measures upheld | As ‘point of reference for what is reasonable’, as indication of public health purpose, as evidentiary support, to outline content of state duties to protect health, to demonstrate ‘internationally accepted’ use of large GHWs, formed basis of WHO/WHO FCTC Secretariat and PAHO amicus curiae briefs |
| 1. BAT v. Secretary of State for Health [2016] EWHC 1169 (Admin) | United Kingdom (High Court of England and Wales) | May 19, 2016 | Standardised (plain) packaging | Intellectual property, property rights and other commercial rights, proportionality of measure, consistency with EU Tobacco Products Directive, extent to which implementing agency considered evidence, powers of implementing agency | Measure upheld | Demonstrating interests at stake, demonstrating proportionality of measure, demonstrating need to exercise caution in relation to tobacco industry evidence |
| 1. Republic of Poland v. European Parliament & Council of the European Union, Case C-358/14 | European Union (European Court of Justice) | May 04, 2016 | EU Tobacco Products Directive (ban on characterizing flavours including menthol) | Powers of implementing agency, commercial rights and interests | Measure upheld | As demonstrating the best available scientific evidence, to demonstrate the proportionality of the measure, supporting inclusion of all flavours in ban |
| 1. R (on the Application of) Philip Morris Brands SARL v. Secretary of State for Health, Case C-547/14 | United Kingdom (European Court of Justice) | May 04, 2016 | EU Tobacco Products Directive (including additive ban, 65% GHWs, partial pack standardization, and bans on cross-border distance sales) | Powers of implementing agency, proportionality, commercial rights | Measure upheld | As demonstrating the best available scientific evidence, to demonstrate the proportionality of the measure, supporting 50+% GHWs |
| 1. Pillbox 38 (UK) Ltd. v. Secretary of State for Health, Case C-477/14 | United Kingdom (European Court of Justice) | May 04, 2016 | EU Tobacco Products Directive (chapter on e-cigarette regulation) | Proportionality, legal basis, discrimination | Measure upheld | Measure noted to implement COP decision on ENDS and therefore to be within discretion of implementing agency |
| 1. British American Tobacco Kenya Ltd. v. Ministry of Health, Petition No. 143 of 2015 | Kenya (High Court) | March 24, 2016 | Tobacco control regulations (including smoke-free laws, tobacco packaging and labelling, disclosure requirements, article 5.3 implementation, and requirements on tobacco companies to contribute 2% of value of products manufactured to a compensation fund) | Public participation rights, due process, legal basis, discrimination, privacy, intellectual property, interpretation of FCTC, proportionality | Regulations upheld, with minor modifications to one of the maximum penalties and a requirement for each company to disclose market share | Legal basis for several provisions, expanding on the right to health, part of the constitutional interpretive framework |
| 1. Philip Morris Asia v. Australia, PCA Case No. 2012-12 | Australia (ad hoc investment tribunal) | December 17, 2015 | Plain (standardised) packaging | Intellectual property, investment law – expropriation, fair and equitable treatment | PM’s challenge dismissed for lack of jurisdiction due to PM’s abuse of right | Objective of measure to implement FCTC. Australia cites FCTC extensively in its defence. |
| 1. Inversiones Eivissa S.A.C. v. Ministry of Health et al, Case No. 3783-2013* | Peru (Lima Superior Court of Justice) | October 05, 2015 | City ordinance defining ‘enclosed public spaces’ for the purposes of the national smoke-free law | Consistency with authorising legislation, powers of implementing agency | Definition of ‘enclosed space’ in ordinance was found to exceed powers of authorising legislation | The authorising law was noted to implement article 8 of the FCTC |
| 1. British American Tobacco Colombia v. Ministry of Health, Case No. 2012-00607-01* | Colombia (State Council) | September 24, 2015 | Ban on certain misleading descriptors | Intellectual property, economic freedoms | Measure upheld | FCTC found to elaborate on the right to life and health and necessary to public interest |
| 1. British American Tobacco of Peru S.A.C. v. Congress of the Republic, Case No. 22881-2010* | Peru (Lima Superior Court of Justice) | July 22, 2015 | Minimum pack size of ten cigarettes | Freedom of enterprise | Measure upheld | Affirms that FCTC is a human rights treaty elaborating the right to health and uses it in proportionality analysis |
| 1. In the matter of Article 122(1)(b) of the Constitution, S.C. (SD) No. 2/2015 | Sri Lanka (Supreme Court) | February 06, 2015 | Legislation implementing 80% graphic health warnings | Intellectual property, freedom of trade | Measure upheld | The legislation is noted to implement article 11 of the FCTC, and the Government relied on articles 2 and 5 of the FCTC to counter arguments that the size of the GHWs was arbitrary |
| 1. British American Tobacco of Peru S.A.C. v. Congress of the Republic, Case No. 22881-2010-0-1801-JR-CI-10* | Peru (Specialised Constitutional Court of Lima) | July 24, 2014 | Minimum pack size of ten cigarettes | Freedom of enterprise | Measure upheld | Affirms that FCTC is a human rights treaty elaborating the right to health and uses it in proportionality analysis |
| 1. British American Tobacco Panama v. Executive Decree No. 611, Docket No. 192 -­11 | Panama (Supreme Court of Justice) | May 28, 2014 | Decree extending legislative ban on tobacco advertising, promotion, and sponsorship to retail display | Freedom of expression, private property, economic freedoms, consistency with authorising legislation | Measure upheld | Implementation of international commitments for health found to be a circumstance which justifies limiting freedom of commercial expression |
| 1. Ceylon Tobacco v. Minister of Health, C.A. 336/2012 | Sri Lanka (Court of Appeal) | May 12, 2014 | Regulations implementing 80% graphic health warnings | Intellectual property, legal basis of measure, consistency with authorising legislation | Upheld but court ordered GHWs to be reduced to 50-60% | Court used the FCTC to interpret authority to require ‘health warnings’ in legislation as including pictorial health warnings |
| 1. Cigarette Distributors Division v. Ministry of Health, HCJ 5975/12* | Israel (Supreme Court) | July 03, 2013 | Vending machine ban | Property, occupational freedom | Measure upheld | Respondent invoked article 13 guidelines to support argument that vending machines constituted TAPS, court found that vending machine ban would reduce TAPS |
| 1. R v. Mader’s Tobacco Store Ltd, 2013 NSPC 29 | Canada (Provincial Court of Nova Scotia) | May 01, 2013 | Retail display ban | Freedom of commercial expression | Measure upheld | FCTC article 13 and guidelines used to demonstrate that retail display bans were a form of advertising, promotion and sponsorship and support the evidence base for the measure |
| 1. JT International SA v. Commonwealth of Australia [2012] HCA 43 | Australia (High Court of Australia) | October 05, 2012 | Plain (standardised) packaging | Constitutional powers to acquire property | Measure upheld | Limited role in litigation, although the court notes that the objective of the measure is to implement FCTC and that this is the basis for the federal government’s power to legislate. |
| 1. Philip Morris Norway v. Health and Care Services of Norway, Civil Action 10-041388TVI-OTIR/02 | Norway (District Court of Oslo) | September 14, 2012 | Retail display ban | Quantitative restrictions under the European Economic Area Agreement | Measure upheld | FCTC Article 13 and guidelines used to demonstrate that measure was suitable and necessary for public health, supported evidence base brought by Norway |
| 1. British American Tobacco South Africa (PTY) Ltd. v. Minister of Health, No. 463/2011 [2012] ZASCA 107 | South Africa (Supreme Court of Appeal) | June 20, 2012 | Comprehensive ban on tobacco advertising, promotion and sponsorship | Freedom of commercial expression | Measure upheld | South Africa’s international obligations relevant to justification of measure, demonstrates practice in open/democratic societies, FCTC relevant to rights to health care and healthy environment |
| 1. Legislative Consultation with Constitutional Division of the Supreme Court, Decision no. 2012-003918 | Costa Rica (Supreme Court) | March 20, 2012 | Tobacco control law comprising a specific tax, prohibition on smoking in enclosed public places, minimum pack sizes and a ban on tobacco advertising, promotion and sponsorship | Powers of legislature, powers of implementing agency, procedural flaws, arbitrariness | Act upheld in entirety | Legal basis for several provisions, demonstrates public health purpose of measure, demonstrates that measures protect fundamental rights to health and life, demonstrates values at stake |
| 1. British American Tobacco of Peru S.A.C. v. Congress of the Republic, Case No. 22881-2010* | Peru (Specialised Constitutional Court of Lima) | January 17, 2012 | Minimum pack size of ten cigarettes | Freedom of enterprise and industry | Measure upheld | Measure implements FCTC article 16; FCTC is a human rights treaty |
| 1. Philip Morris Norway AS v. The Norwegian State, Case E-16/10 | Norway (Court of Justice of the European Free Trade Association States) | September 12, 2011 | Retail display ban | Quantitative restrictions under the European Economic Area Agreement | Returned to national court upon providing an advisory opinion on the relevant legal issues | Parties argued extensively about the status of the WHO FCTC guidelines, EFTA court left these to the national court to resolve |
| 1. 5000 Citizens v. Article 3 of Law No. 28705, Case No. 00032-2010-PI/TC | Peru (Constitutional Court of Peru) | July 19, 2011 | Smoke-free workplaces and public places | Personal autonomy, freedom to run a business | Measure upheld | FCTC is a human rights treaty that gives content to the right to health, measure therefore adopted to fulfil right to health & consistent with constitutional rights |
| 1. R (Sinclair Collis Ltd) v. Secretary of State for Health [2011] EWCA Civ 437 | United Kingdom (Court of Appeal of England and Wales) | June 17, 2011 | Vending machine ban | Quantitative restriction under Treaty on the Functioning of the European Union, property rights | Measure upheld | As evidentiary support; measure noted to implement FCTC articles 13 and 16 and article 13 guidelines’ recommendations to ban vending machines |
| 1. Izmir Association of Coffeehouses v. Prime Minister, Decision No. 2011/8* | Turkey (Constitutional Court) | February 26, 2011 | Smoke-free workplaces and public places | Economic freedoms, property rights, personal autonomy, discrimination | Measure upheld | Measure noted to implement FCTC article 8. |
| 1. Abal Hermanos, S.A. v. Uruguay, Case No. 1713/2010 | Uruguay (Supreme Court of Justice) | November 17, 2010 | 80% graphic health warnings | Powers of implementing agency, intellectual property, economic freedoms, freedom of commercial expression | Measure upheld | Measure noted to implement FCTC article 11 and its guidelines, which provides guidance on effective tobacco control measures |
| 1. Caceres Corrales v. Colombia, Case C-830/2010 | Colombia (Constitutional Court) | October 20, 2010 | Comprehensive ban on tobacco advertising, promotion and sponsorship | Freedom of commercial speech, economic freedoms | Measure upheld | To demonstrate consensus on the serious consequences of tobacco use; as evidence that comprehensive advertising, promotion and sponsorship bans are effective; FCTC noted to elaborate on a number of health-related rights |
| 1. Tabacalera del Este S.A. v. Paraguay, Case No. 754/2010* | Paraguay (Supreme Court) | October 18, 2010 | Regulation providing for smoke-free public places and tobacco packaging and labelling requirements | Powers of implementing agency, consistency with authorising legislation | Regulation found to exceed the power granted under the legislation | FCTC found to be binding on Paraguay but required legislation to implement |
| 1. Amparo Constitucional promovido por el Abog. Marcos Peroni Clifton bajo patrocinio del Aog. Guillermo Peroni, en representación de Philip Morris Paraguay S.A., Case No. 776/2010 | Paraguay (Supreme Court) | October 18, 2010 | Executive decree providing for smoke-free public places and tobacco packaging and labelling requirements | Powers of implementing agency, consistency with authorising legislation | Regulation found to exceed the power granted under the legislation | FCTC found to be binding on Paraguay but required legislation to implement |
| 1. Associação Brasileira de Bares e Restaurantes, seccional São Paulo (ABRASEL-SP) v. Diretor Exectivo da Fundação de Proteção e de Defesa do Consumidor de São Paulo (PROCON-SP), Civil Appeal No. 99010.227637-6.* | Brazil (Court of Justice of São Paulo) | September 13, 2010 | Regulation providing for smoke-free public places and tobacco packaging and labelling requirements | Powers of São Paulo legislature | Measure upheld | FCTC cited as the legal basis of the law |
| 1. Ocampo Uribe v. Colombia, Case C-639/10* | Colombia (Constitutional Court) | August 17, 2010 | Minimum pack size of ten cigarettes | Personal autonomy, solidarity with street sellers | Measure upheld | FCTC demonstrates that tobacco is a health problem and that the measure has a public health aim; measure implements FCTC and therefore legal obligations of the state to protect public health |
| 1. British American Tobacco v. Government of Panama, Case 618-08* | Panama (Supreme Court) | June 03, 2010 | Executive decree providing for smoke-free environments, ban on tobacco advertising, promotion and sponsorship, and enforcement mechanisms | Powers of implementing agency, intellectual property, economic freedoms | Measure upheld | FCTC used to support legal basis for tobacco control measures in view of objective to protect public health, citing Article 8 on smoke-free environments and Article 13 on comprehensive ban on tobacco advertising |
| 1. European Commission v. Republic of France, Case C-197/08 | France (European Court of Justice) | April 04, 2010 | Minimum retail price of cigarettes | Consistency with EU tobacco tax directive | Measure found to be inconsistent with directive | Court notes that France may implement FCTC obligations through the imposition of excise tax instead of minimum pricing |
| 1. Guatemala Chamber of Commerce v. Guatemala, Docket 2158-2009 | Guatemala (Constitutional Court) | February 16, 2010 | Smoke-free law | Freedom of industry and commerce, discrimination, legal certainty | Measure upheld | FCTC used to demonstrate that the measure protects the right to health |
| 1. Unión Tabacalera del Paraguay, et al. v. Paraguay, Case 916/2009* | Paraguay (Supreme Court) | December 28, 2009 | Regulation providing for tobacco packaging and labelling requirements | Powers of implementing agency, consistency with authorising legislation | Regulation found to exceed the power granted under the legislation | FCTC found to be binding on Paraguay but required legislation to implement |
| 1. Miroslav Grcev and Stamen Filipov to the Constitutional Court, Case Nos. 70/2009-0-0; 261/2008-0-0 | Former Yugoslav Republic of Macedonia (Constitutional Court of FYROM) | September 16, 2009 | Smoke-free law | Personal autonomy, economic freedoms | Measure upheld | FCTC cited as one of the instruments demonstrating right to health and public health purpose of measure |
| 1. Agência Nacional de Vigilância Sanitária (ANVISA) v. Souza Cruz S/A, No. 2009.0.01.004853-3* | Brazil (Regional Federal Court of the 2^nd^ Region) | June 17, 2009 | Graphic health warnings | Powers of implementing agency; proportionality – argued images were overly shocking | Measure upheld | As indication of global concern about tobacco and trend to combat it, as indicating need for consumers to be informed. |
| 1. Ministerio Publico Federal v. Agência Nacional de Vigilância Sanitária (ANVISA), No. 2008.72.05.002189-2* | Brazil (Regional Federal Court of the 4^th^ Region) | April 24, 2009 | Graphic health warnings | Right to humane treatment and dignity – argued images were overly shocking and insulting to smokers | Measure upheld | Defence cites FCTC as legal basis for measure |
| 1. Sindicato da Indústria do Fumo no Estado do Rio Grande do Sul v. Agência Nacional de Vigilância Sanitária (ANVISA), No. 2008.04.00.046270-5* | Brazil (Regional Federal Court of the 4^th^ Region) | April 02, 2009 | Graphic health warnings | Powers of implementing agency; proportionality – argued images were overly shocking | Measure upheld | As legal basis; as demonstrating need for and effectiveness of warnings; as expanding on the right to health; supporting proportionality of measure |
| 1. Three Private Individuals v. Baden-Württemberg & Berlin, 1 BvR 3262/07, 1 BvR 402/08; 1 BvR 906/08* | Germany (Federal Constitutional Court) | July 30, 2008 | Smoke-free law | Occupational freedom, discrimination between different types of establishments | Measure declared unconstitutional | FCTC noted as part of the normative background of the law |
| 1. Canada (Attorney General) v. JTI-Macdonald Corp. [2007] 2 S.C.R. 610 | Canada (Supreme Court) | June 28, 2007 | 50% graphic health warnings and ban on ‘lifestyle’ advertising | Freedom of expression | Measure upheld | FCTC cited to support reasonableness of measure and to demonstrate consensus regarding need for 50+% GHWs |
| 1. Ceylon Tobacco Company Ltd.. v. Hon. Nimal Siripala de Silva, S.C. (SD) App. Nos. 1 to 6/2006 | Sri Lanka (Supreme Court) | June 20, 2006 | Smoke-free law | Freedom to engage in a lawful trade, business or enterprise | Measure upheld | FCTC cited as legal basis to protect public health and source of obligation to adopt effective legislative measures to protect from exposure to tobacco smoke |

*No English translation available, information in table based on case summary provided in database in combination with machine translation.
