# Supplementary material for: The impact of the WHO Framework Convention on Tobacco Control in defending legal challenges to tobacco control measures
Source: Tob Control. 2018 Jun 2;28(Suppl 2):s113–8. doi: 10.1136/tobaccocontrol-2018-054329 (PMC6589463; doi:10.1136/tobaccocontrol-2018-054329)
Supplement: Supplementary data [file tobaccocontrol-2018-054329supp002.docx]

**Table 2: legal challenges to regulatory measures in FCTC parties**

| Title | Respondent country / court | Decision date | Measures challenged | Grounds of challenge | Cites FCTC? | Measure upheld? |
| --- | --- | --- | --- | --- | --- | --- |
| 1. B v. Waitemata District Health Board [2017] NZSC 88 | New Zealand (Supreme Court) | June 14, 2017 | Smoke-free policy in hospital/mental health facility | Consistency with other tobacco control legislation; personal autonomy; dignity | n | y |
| 1. British American Tobacco Ltd v. Ministry of Health, CA No. 112 of 2016 (2017) | Kenya (Court of Appeal) | February 17, 2017 | Tobacco control regulations (including smoke-free laws, tobacco packaging and labelling, disclosure requirements, article 5.3 implementation, and requirements on tobacco companies to contribute 2% of value of products manufactured to a compensation fund) | Public participation rights, due process, legal basis, discrimination, privacy, intellectual property, interpretation of FCTC | y | y |
| 1. Japan Tobacco International v. Ministry of Health, Decision Nos. 399117, 399789, 399790, 399824, 399883, 399938, 399997, 402883, 403472, 403823, 404174, 404381, 404394 of 23 December 2016 | France (State Council) | December 23, 2016 | Plain (standardised) packaging | Intellectual property, property rights, consistency with EU Tobacco Products Directive, proportionality, freedom of enterprise, discrimination, legal uncertainty, consistency with procedural rules, authority to impose penalties, powers of implementing agency and legislature | n | y |
| 1. BAT v. Secretary of State for Health [2016] EWCA Civ 1182 | United Kingdom (Court of Appeal of England and Wales) | November 30, 2016 | Standardised (plain) packaging | Intellectual property, proportionality, property and other commercial rights, consistency with EU Tobacco Products Directive | y | y |
| 1. British American Tobacco Panama S.A. s/ Executive Decree 611 of 2010, Docket Nos. 788-10, 818-10, 1013-10. | Panama (Supreme Court) | August 03, 2016 | Decree extending legislative ban on tobacco advertising, promotion, and sponsorship to retail display | Intellectual property, consumer right to receive information | y | y |
| 1. Philip Morris SÀRL v. Uruguay, ICSID Case No. ARB/10/7 | Uruguay (ad hoc investment tribunal) | July 08, 2016 | 80% graphic health warnings, ban on brand variants | Intellectual property, investment law – fair and equitable treatment, expropriation, due process rights, umbrella clause | y | y |
| 1. BAT v. Secretary of State for Health [2016] EWHC 1169 (Admin) | United Kingdom (High Court of England and Wales) | May 19, 2016 | Standardised (plain) packaging | Intellectual property, property rights and other commercial rights, proportionality of measure, consistency with EU Tobacco Products Directive, extent to which implementing agency considered evidence, powers of implementing agency | y | y |
| 1. Republic of Poland v. European Parliament & Council of the European Union, Case C-358/14 | European Union (European Court of Justice) | May 04, 2016 | EU Tobacco Products Directive (ban on characterizing flavours including menthol) | Powers of implementing agency, commercial rights and interests | y | y |
| 1. R (on the Application of) Philip Morris Brands SARL v. Secretary of State for Health, Case C-547/14 | United Kingdom (European Court of Justice) | May 04, 2016 | EU Tobacco Products Directive (including additive ban, 65% GHWs, partial pack standardization, and bans on cross-border distance sales) | Powers of implementing agency, proportionality, commercial rights | y | y |
| 1. Pillbox 38 (UK) Ltd. v. Secretary of State for Health, Case C-477/14 | United Kingdom (European Court of Justice) | May 04, 2016 | EU Tobacco Products Directive (chapter on e-cigarette regulation) | Proportionality, legal basis, discrimination | y | y |
| 1. British American Tobacco Kenya Ltd. v. Ministry of Health, Petition No. 143 of 2015 | Kenya (High Court) | March 24, 2016 | Tobacco control regulations (including smoke-free laws, tobacco packaging and labelling, disclosure requirements, article 5.3 implementation, and requirements on tobacco companies to contribute 2% of value of products manufactured to a compensation fund) | Public participation rights, due process, legal basis, discrimination, privacy, intellectual property, interpretation of FCTC, proportionality | y | y |
| 1. De Bruyn v. Victorian Institute of Forensic Mental Health [2016] VSC 111 | Australia (Supreme Court of Victoria) | March 22, 2016 | Smoke-free policy in mental health facility | Personal autonomy; dignity | n | y |
| 1. Law for the Modernisation of Our Health System, Decision No. 2015-727 DC of 21 January 2016 | France (Constitutional Court) | January 21, 2016 | Amendments to the public health code authorising the adoption of plain (standardised) packaging regulations | Intellectual property, consistency with EU law, property rights, legal certainty | n | y |
| 1. Philip Morris Asia v. Australia, PCA Case No. 2012-12 | Australia (ad hoc investment tribunal) | December 17, 2015 | Plain (standardised) packaging | Intellectual property, investment law – expropriation, fair and equitable treatment | y | y |
| 1. Inversiones Eivissa S.A.C. v. Ministry of Health, Case No. 3783-2013 | Peru (Lima Superior Court of Justice) | October 05, 2015 | Ordinance defining ‘enclosed public spaces’ for the purposes of the smoke-free law | Consistency with authorising legislation, powers of implementing agency | y | n |
| 1. Imperial Tobacco Canada v. Attorney General of Quebec, No. 500-09-024341-141 | Canada (Quebec Court of Appeal) | September 28, 2015 | Health care cost recovery legislation | Due process rights, judicial independence | n | y |
| 1. British American Tobacco Colombia v. Ministry of Health, Case No. 2012-00607-01 | Colombia (State Council) | September 24, 2015 | Ban on certain misleading descriptors | Intellectual property, economic freedoms | y | y |
| 1. British American Tobacco of Peru S.A.C. v. Congress of the Republic, Case No. 22881-2010 | Peru (Lima Superior Court of Justice) | July 22, 2015 | Minimum pack size of ten cigarettes | Freedom of enterprise | y | y |
| 1. City of Vancouver v. Abdolabbas Abdiannia, 2015 BCSC 1058 | Canada (Supreme Court of British Columbia) | June 19, 2015 | Smoke-free law as applied to hookah bars | Application of law to waterpipe tobacco, religious freedom | n | y |
| 1. In the matter of Article 122(1)(b) of the Constitution, S.C. (SD) No. 2/2015 | Sri Lanka (Supreme Court) | February 06, 2015 | Legislation implementing 80% graphic health warnings | Intellectual property, freedom of trade | y | y |
| 1. Narinder S. Chadha v. Municipal Corporation of Greater Mumbai, Civil Appeal No. 10836 of 2014 | India (Supreme Court) | December 08, 2014 | Prohibition on the sale and use of hookah at restaurants | Powers of implementing agency, consistency with authorising legislation | n | n |
| 1. E-Cig Ltd. v. Ministry of Health, HCJ 6665/12 | Israel (Supreme Court) | December 03, 2014 | Administrative requirement for import licences for nicotine | Powers of implementing agency, freedom of occupation | n | n |
| 1. Naya Bans Sarv Vyapar Association v. Union of India, Civil Appeal No. 39271-39272/2012 | India (Supreme Court) | November 11, 2014 | Prohibition on tobacco sales (including wholesale) within 100m of schools | Argued that should be permitted to make wholesale sales near schools if after school hours | n | Previous order allowed wholesale sales after 2pm, time modified to 4pm |
| 1. Charles McCann v. State Hospital Board of Scotland [2014] CSIH 71 | United Kingdom (Court of Session of Scotland, Inner House) | August 12, 2014 | Smoke-free policy in mental health facility | Powers of the implementing agency, privacy, discrimination on the grounds of mental illness, consistency with mental health legislation | n | y |
| 1. British American Tobacco of Peru S.A.C. v. Congress of the Republic, Case No. 22881-2010-0-1801-JR-CI-10 | Peru (Specialised Constitutional Court of Lima) | July 24, 2014 | Minimum pack size of ten cigarettes | Freedom of enterprise | y | y |
| 1. British American Tobacco Panama v. Executive Decree No. 611, Docket No. 192 -­11 | Panama (Supreme Court) | May 28, 2014 | Decree extending legislative ban on tobacco advertising, promotion, and sponsorship to retail display | Freedom of expression, private property, economic freedoms, consistency with authorising legislation | y | y |
| 1. Ceylon Tobacco v. Minister of Health, C.A. 336/2012 | Sri Lanka (Court of Appeal) | May 12, 2014 | Regulations implementing 80% graphic health warnings | Intellectual property, legal basis of measure, consistency with authorising legislation | y | partial |
| 1. Anurag Kashyap v. Union of India, Writ Petition No. 119 of 2014 | India (High Court of Bombay) | May 07, 2014 | Tobacco health warnings in film scenes containing smoking | Freedom of artistic expression | n | y |
| 1. Petition for C M for Judicial Review and Answers for the State Hospitals Board for Scotland [2013] CSOH 143 | United Kingdom (Court of Session of Scotland, Outer House) | August 27, 2013 | Smoke-free policy in mental health facility | Powers of the implementing agency, privacy, discrimination on the grounds of mental illness, consistency with mental health legislation | n | n |
| 1. B v. Waitemata District Health Board [2013] NZHC 1702 | New Zealand (High Court) | July 08, 2013 | Smoke-free policy in hospital / mental health facility | Administrative decisionmaking, due process rights, discrimination on the grounds of mental illness and against smokers, privacy, torture/cruel treatment | n | y |
| 1. Cigarette Distributors Division v. Ministry of Health, HCJ 5975/12 | Israel (Supreme Court) | July 03, 2013 | Vending machine ban | Property, occupational freedom | y | y |
| 1. Taylor v. Attorney General [2013] NZHC 1659 | New Zealand (High Court) | July 03, 2013 | Smoke-free regulations in prisons | Powers of implementing agency, consistency with corrections and smoke-free legislation | n | n |
| 1. R v. Mader’s Tobacco Store Ltd, 2013 NSPC 29 | Canada (Provincial Court of Nova Scotia) | May 01, 2013 | Retail display ban | Freedom of commercial expression | y | y |
| 1. Naya Bans Sarv Vyapar Association v. Union of India, Civil Appeal No. 39271-39272/2012 | India (Supreme Court) | February 01, 2013 | Prohibition on tobacco sales (including wholesale) within 100m of schools | Argued that should be permitted to make wholesale sales near schools if after school hours | n | Previous order modified to allow wholesale sales after 2pm |
| 1. Taylor v. Manager of Auckland Prison [2012] NZHC 3591 | New Zealand (High Court) | December 20, 2012 | Smoke-free regulations in prisons | Powers of implementing agency, consistency with corrections and smoke-free legislation | n | n |
| 1. Imperial Tobacco v. Lord Advocate (Scotland) [2012] UKSC 61 | United Kingdom (Supreme Court) | December 12, 2012 | Vending machine bans | Powers of Scottish legislature | n | y |
| 1. Naya Bans Sarv Vyapar Association v. India, W.P. No.7292/2011 | India (High Court of Delhi) | November 09, 2012 | Prohibition on tobacco sales within 100m of schools | Argued that law should not be applied to wholesale sales | n | y |
| 1. Sinclair Collis Ltd. v. Lord Advocate for Scotland [2012] CSIH 80 | United Kingdom (Inner House of the Court of Session of Scotland) | October 10, 2012 | Vending machine ban | Quantitative restriction under Treaty on the Functioning of the European Union | n | y |
| 1. JT International SA v. Commonwealth of Australia [2012] HCA 43 | Australia (High Court of Australia) | October 05, 2012 | Plain (standardised) packaging | Constitutional powers to acquire property | y | y |
| 1. Philip Morris Norway v. Health and Care Services of Norway, Civil Action 10-041388TVI-OTIR/02 | Norway (District Court of Oslo) | September 14, 2012 | Retail display ban | Quantitative restrictions under the European Economic Area Agreement | y | y |
| 1. British American Tobacco South Africa (Pty) Limited v. Minister of Health, Case CCT 65/12 | South Africa (Constitutional Court) | August 06, 2012 | Comprehensive ban on tobacco advertising, promotion and sponsorship | Freedom of commercial expression | n | Y (appeal dismissed at summary stage as no prospects of success) |
| 1. Yadav v. State of Bihar, CWJC No. 10297 of 2012 | India (High Court of Patna) | July 10, 2012 | Order banning the production of gutka/pan masala containing tobacco | Consistency with authorising legislation, powers of implementing agency | n | y |
| 1. British American Tobacco South Africa (Pty) Ltd. v. Minister of Health, No. 463/2011 [2012] ZASCA 107 | South Africa (Supreme Court of Appeal) | June 20, 2012 | Comprehensive ban on tobacco advertising, promotion and sponsorship | Freedom of commercial expression | y | y |
| 1. Parramatta Business Freedom Association Inc v. Parramatta City Council [2012] NSWLEC 139 | Australia (New South Wales Land and Environment Court) | June 20, 2012 | Local council regulation banning smoking in outdoor dining areas | Powers of the local council, exercise of discretion | n | n |
| 1. Legislative Consultation with Constitutional Division of the Supreme Court, Decision no. 2012-003918 | Costa Rica (Supreme Court) | March 20, 2012 | Tobacco control law comprising a specific tax, prohibition on smoking in enclosed public places, minimum pack sizes and a ban on tobacco advertising, promotion and sponsorship | Powers of legislature, powers of implementing agency, procedural flaws, arbitrariness | y | y |
| 1. Imperial Tobacco Limited v. The Lord Advocate, The Scottish Ministers [2012] CSIH 9 | United Kingdom (Court of Session of Scotland, Inner House) | February 02, 2012 | Retail display and vending machine bans | Whether Scottish legislature had power to adopt law; consistency with UK national tobacco control legislation | n | y |
| 1. British American Tobacco of Peru S.A.C. v. Congress of the Republic, Case No. 22881-2010 | Peru (Specialised Constitutional Court of Lima) | January 17, 2012 | Minimum pack size of ten cigarettes | Freedom of enterprise and industry | y | y |
| 1. Philip Morris Norway AS v. The Norwegian State, Case E-16/10 | Norway (Court of Justice of the European Free Trade Association States) | September 12, 2011 | Retail display ban | Quantitative restrictions under the European Economic Area Agreement | y | Remanded to domestic court |
| 1. Berrys Hotel (MOCHA) v. Municipal Corporation of Greater Mumbai, W.P. (L) No. 1531-2011 | India (High Court of Bombay) | August 11, 2011 | Prohibition on the sale of tobacco as a condition of restaurant licences | Power to impose licence conditions, consistency with authorising legislation | n | y |
| 1. 5000 Citizens v. Article 3 of Law No. 28705, Case No. 00032-2010-PI/TC | Peru (Constitutional Court of Peru) | July 19, 2011 | Smoke-free workplaces and public places | Personal autonomy, freedom to run a business | y | y |
| 1. R (Sinclair Collis Ltd) v. Secretary of State for Health [2011] EWCA Civ 437 | United Kingdom (Court of Appeal of England and Wales) | June 17, 2011 | Vending machine ban | Quantitative restriction under Treaty on the Functioning of the European Union, property rights | y | y |
| 1. British American Tobacco South Africa (Pty) Ltd v. Minister of Health, Case 60230/2009 | South Africa (High Court) | May 19, 2011 | Comprehensive ban on tobacco advertising, promotion and sponsorship | Freedom of commercial expression | n | y |
| 1. Sinclair Collis Limited v. Lord Advocate for Scotland [2011] CSOH 80 | United Kingdom (Outer House of the Court of Session of Scotland) | May 13, 2011 | Vending machine ban | Quantitative restriction under the Treaty on the Functioning of the European Union | n | y |
| 1. Izmir Association of Coffeehouses v. Prime Minister, Decision No. 2011/8 | Turkey (Constitutional Court) | February 26, 2011 | Smoke-free workplaces and public places | Economic freedoms, property rights, personal autonomy, discrimination | y | y |
| 1. Abal Hermanos, S.A. v. Uruguay, Case No. 1713/2010 | Uruguay (Supreme Court of Justice) | November 17, 2010 | 80% graphic health warnings | Powers of implementing agency, intellectual property | y | y |
| 1. Thailand – Customs and Fiscal Measures on Cigarettes from the Philippines, Panel Report, DS 371 | Thailand (WTO Panel) | November 15, 2010 | Various fiscal and tax measures applied to imported cigarettes | Discrimination between imported and domestic cigarettes | n | n |
| 1. Caceres Corrales v. Colombia, Case C-830/2010 | Colombia (Constitutional Court) | October 20, 2010 | Comprehensive ban on tobacco advertising, promotion and sponsorship | Freedom of commercial speech, economic freedoms | y | y |
| 1. Tabacalera del Este S.A. v. Paraguay, Case No. 754/2010 | Paraguay (Supreme Court) | October 18, 2010 | Regulation providing for smoke-free public places and tobacco packaging and labelling requirements | Powers of implementing agency, consistency with authorising legislation | y | n |
| 1. Amparo Constitucional promovido por el Abog. Marcos Peroni Clifton bajo patrocinio del Aog. Guillermo Peroni, en representación de Philip Morris Paraguay S.A., Case No. 776/2010 | Paraguay (Supreme Court) | October 18, 2010 | Executive decree providing for smoke-free environments, ban on tobacco advertising, promotion and sponsorship, and enforcement mechanisms | Due process, legal basis | y | n |
| 1. Imperial Tobacco Ltd., Re Judicial Review [2010] CSOH 134 | United Kingdom (Outer House of the Court of Session of Scotland) | September 30, 2010 | Retail display and vending machine bans | Powers of legislature, interference with freedom to trade within UK | n | y |
| 1. Associação Brasileira de Bares e Restaurantes, seccional São Paulo (ABRASEL-SP) v. Diretor Exectivo da Fundação de Proteção e de Defesa do Consumidor de São Paulo (PROCON-SP), Civil Appeal No. 99010.227637-6 | Brazil (Court of Justice of São Paulo) | September 13, 2010 | Regulation providing for smoke-free public places and tobacco packaging and labelling requirements | Powers of São Paulo legislature | y | y |
| 1. British American Tobacco Central América, S.A. v. Guatemala, Docket No. 1183-2009 | Guatemala (Constitutional Court) | September 02, 2010 | Additional tax rate on imported cigarettes | Argued that imported cigarettes were taxed more than domestic cigarettes | n | n |
| 1. R v. Mader's Tobacco Store Ltd, 2010 NSPC 52 | Canada (Provincial Court of Nova Scotia) | August 18, 2010 | Retail display ban | Freedom of commercial expression | n | N but successfully appealed |
| 1. Ocampo Uribe v. Colombia, Case No. C-639/10 | Colombia (Constitutional Court) | August 17, 2010 | Minimum pack size of ten cigarettes | Personal autonomy, solidarity with street sellers | y | y |
| 1. Correctional Service of Canada v. Mercier, 2010 FCA 167 | Canada (Federal Court of Appeals) | June 21, 2010 | Smoking ban in prisons | Powers of implementing agency | n | y |
| 1. British American Tobacco v. Government of Panama, Docket No. 618-08 | Panama (Supreme Court) | June 03, 2010 | Executive decree providing for smoke-free environments, ban on tobacco advertising, promotion and sponsorship, and enforcement mechanisms | Regulation exceeds the powers granted by legislation, right to use trademarks and to conduct business activity | y | y |
| 1. Decision No. 2010-040 of 22 April 2010 | Belgium (Constitutional Court) | April 22, 2010 | Smoke-free indoor dining areas (with exemption for drinking establishments serving only packaged foods with an expiry date of 3+ months) | Discrimination between establishments serving different types of food | n | y |
| 1. European Commission v. Republic of France, Case C-197/08 | France (European Court of Justice) | April 04, 2010 | Minimum retail price of cigarettes | Consistency with EU tobacco tax directive | y | n |
| 1. Guatemala Chamber of Commerce v. Guatemala, Docket No. 2158-2009 | Guatemala (Constitutional Court) | February 16, 2010 | Smoke-free workplaces and public places | Freedom of industry and commerce, discrimination, legal certainty | y | y |
| 1. Unión Tabacalera del Paraguay v. Paraguay, Case No. 916/2009 | Paraguay (Supreme Court) | December 28, 2009 | Regulation providing for tobacco packaging and labelling requirements | Powers of implementing agency, consistency with authorising legislation | y | n |
| 1. British American Tobacco v. Tobacco and Alcohol Markets Regulatory Authority, Decision No. 2009/5805 | Turkey (State Council) | December 01, 2009 | By-law giving authority to regulate packaging, 65% graphic health warnings (GHWs) | Powers of implementing agency, consistency with other tobacco control legislation | n | Authority to regulate upheld, but GHWs found to be inconsistent with other laws requiring smaller warnings |
| 1. Mercier v. Correctional Service of Canada, 2009 FC 1071 | Canada (Federal Court) | October 23, 2009 | Smoking ban in prisons | Powers of implementing agency | n | N but successfully appealed |
| 1. Miroslav Grcev and Stamen Filipov to the Constitutional Court, Case Nos. 70/2009-0-0; 261/2008-0-0 | Former Yugoslav Republic of Macedonia (Constitutional Court of FYROM) | September 16, 2009 | Smoke-free law | Personal autonomy, economic freedoms | y | y |
| 1. R (N) v. Secretary of State for Health [2009] EWCA Civ 795 | United Kingdom (Court of Appeal of the Supreme Court of Judicature) | July 24, 2009 | Smoking ban in mental health facilities | Right to privacy in the home, discrimination against mentally ill persons | n | y |
| 1. Winsa S.A. v. Mexico, No. 167118 | Mexico (Collegiate Circuit Courts) | June 29, 2009 | Smoking ban in indoor dining areas | Discrimination | n | y |
| 1. Hamdan Amad, Faudi v. Mexico, No. 167087 | Mexico (Collegiate Circuit Courts) | June 29, 2009 | Smoking ban in enclosed public places | Personal autonomy | n | y |
| 1. Agência Nacional de Vigilância Sanitária (ANVISA) v. Souza Cruz S/A, No. 2009.0.01.004853-3 | Brazil (Regional Federal Court of the 2^nd^ Region) | June 17, 2009 | Graphic health warnings | Powers of implementing agency; proportionality – argued images were overly shocking | y | y |
| 1. Desert Oasis Company v. Republic of France, Decision No. 318066 of 10 June 2009. | France (State Council) | June 10, 2009 | Smoke-free law | Powers of implementing agency | n | y |
| 1. Ministerio Publico Federal v. Agência Nacional de Vigilância Sanitária (ANVISA), No. 2008.72.05.002189-2 | Brazil (Regional Federal Court of the 4^th^ Region) | April 24, 2009 | Graphic health warnings | Right to humane treatment and dignity – argued images were overly shocking and insulting to smokers | y | y |
| 1. Sindicato da Indústria do Fumo no Estado do Rio Grande do Sul v. Agência Nacional de Vigilância Sanitária (ANVISA), No. 2008.04.00.046270-5 | Brazil (Regional Federal Court of the 4^th^ Region) | April 02, 2009 | Graphic health warnings | Powers of implementing agency; proportionality – argued images were overly shocking | y | y |
| 1. Operadora de Centros de Espectáculos, S.A. de C.V. v. Mexico, No. 168130 | Mexico (Collegiate Circuit Courts) | January 29, 2009 | Prohibition on the sale of tobacco in entertainment venues | Right to commerce | n | n |
| 1. Mahesh Bhatt v. Union of India, Writ Petition (Civil) No. 18761 of 2005 | India (High Court of Delhi) | January 23, 2009 | Regulations prohibiting display of tobacco products in television and film | Freedom of artistic expression, powers provided under authorising legislation | n | n |
| 1. National Union of Environmental Health Personnel v. France, Decision No. 303937 of 8 October 2008 | France (State Council) | October 08, 2008 | Authority to certain agencies to enforce law | Exceeding powers of authorising legislation | n | y |
| 1. Comercial Hotelera Mexicana de Occidente, S.A. v. Mexico, No. 168757 | Mexico (Collegiate Circuit Courts) | September 28, 2008 | Prohibition on the sale of tobacco products in hotels and restaurants | Discrimination between hotels/restaurants and other entertainment venues | n | n |
| 1. N v. Bavaria, 1BvR 3198/07 | Germany (Federal Constitutional Court) | August 06, 2008 | Ban on smoking in restaurants | Freedom of occupation, personal autonomy | n | y |
| 1. Three Private Individuals v. Baden-Württemberg & Berlin, 1 BvR 3262/07, 1 BvR 402/08; 1 BvR 906/08 | Germany (Federal Constitutional Court) | July 30, 2008 | Smoke-free law | Occupational freedom, discrimination between different types of establishments | y | n |
| 1. R (G) v. Nottinghamshire Healthcare NHS Trust [2008] EWHC 1096 | United Kingdom (High Court of Justice of England and Wales) | May 20, 2008 | Ban on smoking in mental health facilities | Right to privacy in the home, discrimination on the basis of mental illness | n | y |
| 1. Mahesh Bhatt and Kasturi and Sons v. Union of India, Writ Petition (Civil) Nos. 18761, 23716/2005 and 7410-11/2006 | India (High Court of Delhi) | February 07, 2008 | Legislation prohibiting display of tobacco products in films | Freedom of artistic expression | n | y |
| 1. Boucher v. Attorney General of Canada, 2007 FC 893 | Canada (Federal Court) | September 07, 2007 | Ban on smoking in prisons | Powers of implementing agency, consistency with authorising legislation | n | y |
| 1. Canada (Attorney General) v. JTI-Macdonald Corp. [2007] 2 S.C.R. 610 | Canada (Supreme Court) | June 28, 2007 | 50% graphic health warnings and ban on ‘lifestyle’ advertising | Freedom of expression | y | y |
| 1. Mme B. v. Republic of France, Decision No. 300467 of 19 March 2007 | France (State Council) | March 19, 2007 | Smoke-free law | Right to property, discrimination against smokers, freedom of assembly, personal autonomy | n | y |
| 1. M.Y. v. the Republic of France, Decision Nos. 300467, 300500, 300680, 300681, 300682, 300683, 300898 of 19 March 2007 | France (State Council) | March 19, 2007 | Smoke-free decree | Discrimination against smokers, powers of implementing agency, personal autonomy, freedom of assembly | n | y |
| 1. Robin v. France, Decision No. 0400326 of 28 December 2006 | France (Administrative Court of Caen) | December 28, 2006 | Smoke-free decree | Consistency with authorising legislation | n | Read down to exclude private offices |
| 1. Ceylon Tobacco Company Ltd. v. Hon. Nimal Siripala de Silva, S.C. (SD) App. Nos. 1 to 6/2006 | Sri Lanka (Supreme Court) | June 20, 2006 | Smoke-free law | Freedom to engage in a lawful trade, business or enterprise | y | y |
| 1. The Lion Brewery Ceylon Ltd. v. Hon. Attorney General, S.C. (SD) Nos. 13-22/05 | Sri Lanka (Supreme Court) | January 16, 2006 | National Authority on Tobacco and Alcohol (NATA) Bill which creates NATA and providing it with certain powers; and introduces a ban on tobacco and alcohol advertising, promotion, and sponsorship | Discrimination, freedom of commercial expression | n | y |
| 1. British Columbia v. Imperial Tobacco Canada, [2005] 2 S.C.R. 473 | Canada (Supreme Court | September 29, 2005 | Health care cost recovery | Judicial independence, due process rights | n | y |
